# Policy learning and Universal Health Coverage in low- and middle-income countries

**DOI:** 10.1186/s12961-020-00591-z

**Published:** 2020-07-21

**Authors:** Joël Arthur Kiendrébéogo, Manuela De Allegri, Bruno Meessen

**Affiliations:** 1 Department of Public Health, Health Sciences Training and Research Unit, University Joseph Ki-Zerbo, Ouagadougou, Burkina Faso; 2 Department of Public Health, Institute of Tropical Medicine, Antwerp, Belgium; 3 Heidelberg Institute of Global Health, Medical Faculty and University Hospital, Heidelberg University, Heidelberg, Germany; 4 Health Systems Governance and Financing, WHO, Geneva, Switzerland

**Keywords:** Health policy, Learning, Knowledge, Universal Health Coverage, Low- and middle-income countries

## Abstract

Learning is increasingly seen as an essential component to spur progress towards universal health coverage (UHC) in low- and middle-income countries (LMICs). However, learning remains an elusive concept, with different understandings and uses that vary from one person or organisation to another. Specifically, it appears that ‘learning for UHC’ is dominated by the teacher mode — notably scientists and experts as ‘teachers’ conveying to local decision/policy-makers as ‘learners’ what to do. This article shows that, to meet countries’ needs, it is important to acknowledge that UHC learning situations are not restricted to the most visible epistemic learning approach practiced today. This article draws on an analytical framework proposed by Dunlop and Radaelli, whereby they identified four learning modes that can emerge according to the specific characteristics of the policy process: epistemic learning, learning in the shadow of hierarchy, learning through bargaining and reflexive learning. These learning modes look relevant to help widen the learning prospects that LMICs need to advance their UHC agenda. Actually, they open up new perspectives in a research field that, until now, has appeared scattered and relatively blurry.

## Background

Advancing universal health coverage (UHC) to improve population health is a long-term objective that many low- and middle-income countries (LMICs) have committed to. Since the release of the World Health Report 2010, entitled ‘Health systems financing: the path to universal coverage’ [1] — the flagship document that popularised the concept — extensive research and initiatives have focused on the subject matter at national and international level. Such research and initiatives have helped to map the many challenges, identify best approaches to spur countries’ progression [2, 3]; assess progress made by countries and build databases describing the situation prevailing in countries [4]. Yet, in many countries, challenges remain. Research findings are not always properly integrated into policy and practice [5]. Above all, UHC is a complex endeavour at the crossroads of technique and politics, applied to health systems that are themselves complex and, thus, to some extent unpredictable [6, 7]. For instance, a policy that has succeeded in one place may fail in another [8] or the results of a policy designed and implemented in a country may be quite the opposite of what was expected [9]. Therein, it has been argued that the ability to continually learn and adapt is essential — learning should then be at the heart of UHC-related policy processes [10–12]. Indeed, there is growing interest among global health actors towards ‘learning for UHC’. Some of them even have an explicit learning-oriented mandate in their support to countries such as the ‘Joint Learning Network for UHC’, ‘P4H Network’, ‘UHC partnership’ and ‘UHC 2030′.

Yet, there is not much scientific literature on learning processes related to UHC [13] and questions abound, some highlighted in Table 1. These questions, to a large extent, remain either not answered or only partially answered, probably because learning, itself, is an elusive concept — it is framed, defined, understood and used differently from one person or organisation to another [14]. This article is not intended to provide a specific answer to each of these questions but to enrich our knowledge and understanding of what ‘learning for UHC’ could entail. It is worth mentioning that, if the attention to learning is relatively new in health policy, the concept has a long tradition in academia and has been extensively studied in other disciplines such as psychology, education, international relations, sociology, organisational studies and political science [15].

**Table 1:** Examples of relevant questions related to ‘learning for UHC’

• How does 'learning for UHC' occur at country level?	
• What type(s) of learning predominate or are favoured at country level and why?	
• What is the role of learning in policy-making processes?	
• What dynamics (actors and factors) facilitate or hinder learning processes at country level? Specifically, how does context, including organisations’ features and dynamics, shape learning and affect learning outcomes?	
• How and by whom are countries’ learning needs identified? Are they properly identified?	
• What actions are being taken to address these needs? Are they successful?	

Learning can be approached through different theoretical and pragmatic perspectives, the most prominent ones including cognitivism, behaviourism and constructivism [16], or even social constructionism if we add a social dimension [17]. From the cognitive stance, learning is related to the acquisition of new insights, assumptions, understandings and awareness resulting in new mental models or belief systems [18]. The behavioural stance, meanwhile, insists on the need that such cognitive changes be followed, simultaneously or after, by ‘shifts in actions or behaviours’ — the so-called ‘cognitive-behavioural perspective’ [18]. Changes in actions or behaviours in turn influence the cognitive aspects of learning in a kind of iterative loop [19], as observed in the ‘action learning’, ‘after action review’, ‘action research’ and ‘learning-by-doing’ approaches. As for the social constructionism stance, learning emerges from social interactions and realities through formal and informal networks such as communities of practice defined as “*groups of people who share a concern, set of problems, or a passion about a topic, and who deepen their knowledge and expertise in this area by interacting on an ongoing basis*” ([20], p. 4); for instance, when young practitioners learn by interacting with experienced medical staff in a hospital [21].

This paper adopts and adapts the definition of learning put forward by two political scientists, Dunlop and Radaelli [14] — learning is the updating of knowledge, beliefs and actions based on lived or witnessed experiences, analysis or social interaction. Beyond its synthetic nature, this definition meets our special interest for public policies, specifically UHC-related policy processes.

The political science literature provides us with the concept of ‘policy learning’, which is learning applied to policy-making processes. It “*occurs through the very practice of policy-making*” ([22], p. 273). Moyson and Scholten define it as “*the cognitive and social dynamic leading policy actors to revise or strengthen their policy beliefs and preferences over time*” ([23], p. 27). Policy learning can manifest itself in a variety of ways, notably *“as updates to our understanding of instrumental or technical aspects of a policy problem, as changes to our underlying policy beliefs or values about societal priorities in responding to problems, and as fundamental alterations to the institutions that target these problems*” [24] and also as adoption of new and innovative ideas.

In the next sections of this paper, we first give a (non-exhaustive) overview of the literature on learning and how we came up to adopt the analytical framework by Dunlop and Radaelli [14]. Thereafter, we critically reflect on how this framework could help LMICs widen the learning prospects they need to advance their UHC agenda. Actually, without claiming to be exhaustive, we find this framework relevant to account for and capture a multitude of learning situations encountered empirically during UHC processes. These learning situations (or learning modes) could serve as reference points for national and international actors engaged in promoting learning for UHC to gain more insights on what they are doing and to help them make deep analyses and critical reflections on their actions in order to improve them.

### Literature review and methodological considerations

Navigating the literature on policy learning is a daunting task since the latter is ‘characterised by concept stretching’ [14, 25, 26] and resembles a maze where the risk of straying is ever present. This is exemplified by a recent bibliometric study conducted by Goyal and Howlett [25], which identified 547 publications on the topic from 1976 to 2016, and other literature reviews performed by leading scientists in the field [23, 27, 28]. Actually, the taxonomy of learning is rich, depending for instance on the content, direction and framing of learning [28] or the methods and tools used. Hence, learning types are diverse and not necessarily mutually exclusive, including, among others, instrumental learning, social learning, political learning (May [29], Hall [30]), policy-oriented learning (Sabatier [31]), government learning (Etheredge and Short [32]) and organisational learning (Argyris and Schön [19]). Besides, other concepts are closely linked to learning such as those of policy transfer (Dolowitz and Marsh [33]), policy diffusion (Shipan and Volden [34], Marsh and Sharman [35]), policy convergence (Bennett [36], Holzinger and Knill [37]) and lesson drawing (Rose [38]).

In general, policy learning is studied in relation to policy change and fits best into the large group of cognitive approaches to public policy analysis, a school of thought that emphasises the role of ideas, beliefs, values and norms in public policy [39]. Actually, before Heclo (1974) [40], the hitherto dominant paradigm was that only conflicts and power relations convincingly explain changes in public policy. Heclo [40] and followers of his school of thought challenge such prospect and emphasise the crucial role of ideas and learning. Indeed, Heclo argues that “*politics finds its sources not only in power, but also in uncertainty – men collectively wondering what to do*” ([40], p. 305); learning is thus seen as an answer to “*the problem of managing and reducing radical uncertainty*” ([27], p. 3) in policy-making. Then, policy learning somehow opens perspectives in the analysis and understanding of complex interactions between knowledge, policy and power [41].

Moreover, it has been postulated that learning does not only generate positive effects and could have its setbacks. Indeed, learning is not risk-free if one does not rely on the right actors, if its content is poorly understood and/or if its goals are diverted. For instance, it may happen that one is “*persevering in listening to the wrong teachers*”, “*implementing the wrong lesson*” or “*applying the right lesson to the wrong institutional context*” ([27], p. 1), especially if there are no self-critical processes and/or iterative learning loops. Furthermore, if learning purposes are ill-defined or poorly specified, it can be manipulated and used to legitimise choices already made and/or serve private or hidden interests [27]. Finally, as usual in any policy process [42], learning and its effects on subsequent policies can have political, economic or social implications, with vested interests of major players at stake. It is therefore important to analyse and consider the political economy surrounding learning endeavours [43, 44].

By delving into the political science literature on learning, a book chapter has particularly attracted our interest since it was helpful in navigating the vast literature on ‘policy learning’. This chapter concerns the allegorical description by Dunlop et al. [27] in the form of a family tree, of the evolution of the concept from the founding fathers (notably John Dewey, Harold Lasswell, Karl Deutsch, Charles Lindblom, Herbert Simon and Hugh Heclo) to the most recent developments. Dunlop et al. [27] distinguish three main periods: the late 1920s to the 1990s (corresponding to the roots of ‘policy learning’), the 1990s to the 2010s (assimilated to the trunk of the tree) and 2010s to the present (representing the branches of the tree). They assert that recent work is “*less concerned with the type of learning per se (instrumental, political, social …) and more focused on the characteristics of the policy process that determine varieties or modes of learning*” ([27], p. 11). For example, the policy process can hold epistemic, hierarchical, bargaining-oriented or reflexive trait [27]. This strong connection between learning features and policy process features resonated with recent work that Dunlop and Radaelli have pioneered, sparking our interest in the analytical framework they proposed [14].

Dunlop and Radaelli [14] use the ‘concept formation’ approach proposed by Sartori [45] and the ‘exploratory typologies’ technique described by Elman [46] to make the concept of policy learning more tangible. For that, they identify, from the literature, two main dimensions that matter in the social and learning mechanisms of policy processes. The first one is ‘problem tractability’, which relates to the level of uncertainty regarding the policy issue under discussion, the degree of solvency of the problems subject to learning [28] — “*a repertoire of solutions, algorithms, or ways of doing things*” exists ([47], p. 261). Low tractability is equivalent to high uncertainty and vice versa. When tractability is high, the transferability and diffusion of lessons learned and solutions from one setting to another is easier, and vice versa. The second one is ‘actors’ certification’, that refers to “*the authority and legitimacy of some key actors or venues*” ([14], p. 602) — certified actors have a privileged position to influence decision/policy-making and the higher their level of certification, the higher this privilege. Drawing on adult education science, Dunlop and Radaelli [14] metaphorically assimilate ‘learners’ to decision/policy-makers or policy implementers and ‘teachers’ to knowledge holders or producers (e.g. experts, scientists, interest groups, think tanks) striving to influence decision/policy-making or institutional rules. In this perspective, low actors’ certification equates to a low divide between the learner and the teacher — there is no knowledge hierarchy.

**Fig. 1:**
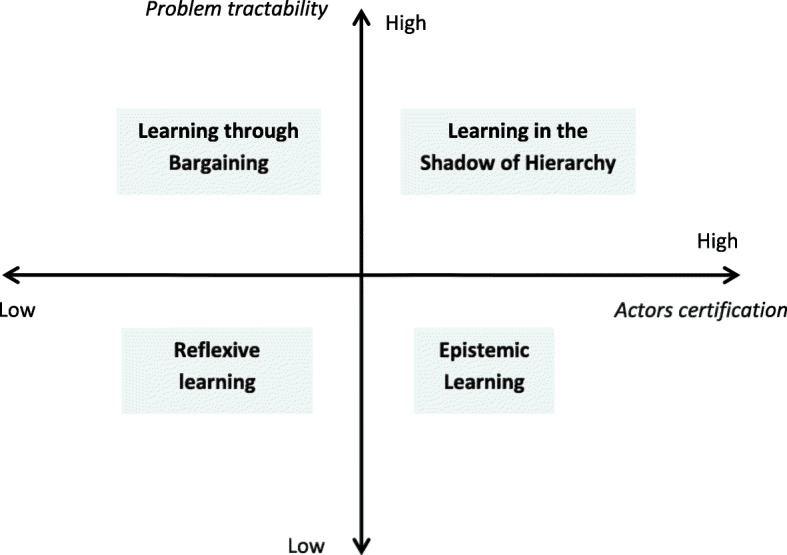
The four modes of policy learning. Source: Adapted from Dunlop and Radaelli [14]

By crossing these two dimensions, ‘problem tractability’ and ‘actors’ certification’, Dunlop and Radaelli [14] end up with a four-quadrants matrix and subsequently classify the vast literature on policy learning according to these four quadrants. In doing so, they identify four learning modes — epistemic, reflexive, bargaining and hierarchical learnings — depending on the level of uncertainty or actors’ certification vis-à-vis the policy issue (Fig. 1). We postulate that these four learning modes could help better understand the scope and variety of configurations that ‘learning for UHC’ can take.

Epistemic learning and learning in the shadow of hierarchy, typically, are vertical and prescriptive ways of learning. In epistemic learning, you have (1) someone who ‘knows’ and someone who is likely to learn, (2) intractable policy issues looking for technocratic answers. ‘Expert power’ [48] is actually used to look for solutions to well-identified problems. Experts and scientists are at the heart of the policy process and enlighten policy-making through their authoritative knowledge. As for learning in the shadow of hierarchy, it piggybacks on the exercise of authority, such as a principal who creates some pressure on an agent to learn [14], for example, because of frequent supervisions. Such learning may be used to achieve specific or predefined goals or results. If epistemic learning and learning in the shadow of hierarchy are two vertical learning modes, reflexive learning and learning through bargaining are rather horizontal — there is no pecking order in knowledge. Reflexive learning entails open, deep, inclusive and critical discussions without (self) censorship between policy actors to gain mutual meaningful insights on issues at stake. Learning through bargaining, meanwhile, implies repeated social interactions and “*is often the unintended product of dense systems of interaction between politicians and bureaucrats*” ([14], p. 604).

### Applying Dunlop and Radaelli’s framework to UHC processes in LMICs

Dunlop and Radaelli’s approach to policy learning modes enriches the field of public policy analysis by highlighting the role of learning in policy-making and decision-making spaces, both conceptually and empirically. This is relevant from the UHC perspective regarding the critical role that learning could play in UHC processes, especially with the complexity of health systems [6, 7]. Our hypothesis is that Dunlop and Radaelli’s work offers an opportunity to pursue a reflection in this direction, starting with the learning modes they propose. In the next sections, we offer some personal reflections on how these learning modes already contribute and could probably be even more applied to UHC in LMICs. Due to our professional history, we are probably privier or more acquainted to epistemic forms of learning through our own engagement in epistemic communities. However, where appropriate, we strived to ignore this posture and took the critical distance needed to explore/illustrate the other forms or modes of learning occurring in UHC processes. When relevant, we also highlight how learning intertwines with power relationships.

#### The case of epistemic learning

Epistemic learning is probably the most visible, analysed and rationalised mode of learning today in global health [49]. This is probably also true for the UHC agenda, something which might be explained by its strong technical dimension and health financing lineage [1, 50]. When we are facing a question, we look for an expert or an actor able to implement a rigorous approach to remove the uncertainty. It is so prevailing that it is actually our main understanding or expectation of how learning should take place — this seems particularly true within a scientific community so committed to research and the prospect of evidence-informed policy. Epistemic learning takes several configurations in our ‘collective action for UHC’ in LMICs. Epistemic learning encompasses situations such as (1) reading a policy-brief or even a scientific article, particularly a systematic review or a meta-analysis; (2) attending national, regional or international meetings or training workshops; (3) the release of conceptual or analytical frameworks to better understand the concept of UHC or its linkages with health system pillars — examples include the health financing functions [50, 51] or the ‘UHC cube’ [1]; or (4) specialist agencies or researchers sharing lessons learned in other countries [3, 52] or developing policy guidance notes on how to move quickly towards UHC [53–55].

Epistemic learning can also take a more active form, for instance, as technical assistance to countries in various possible arrangements — ‘fly-in/fly-out’ or long-term technical assistance [56], with local and/or international experts who are embedded or not in government institutions, and acting as individuals or as part of national, bi-multilateral or international bodies. In any case, their mandate would be to assist countries implementing complex reforms or still struggling to find the ‘right’ policies tailored to their context and/or the proper way to design and implement them, including institutional arrangements and policy instruments [57]. Examples include how to improve healthcare services utilisation and quality, how to improve public financial management or how to make health care services purchasing more strategic. Decision/policy-makers are thus expected to rely on the knowledge of scientists and experts, sometimes in the form of a coaching or mentoring approach [56] to find solutions to these intractable issues.

Ideally, these scientists and experts should be people familiar with the technical and non-technical (e.g. political, social, cultural, economic) intricacies of the context — a condition not always fulfilled [58, 59]. They would then have both legitimate, expert and informational power [48] to advise countries or technical departments of Ministries in charge of UHC on how to successfully implement specific policies or processes, taking into account path-dependency and other local specificities [60]. However, sometimes, even deep contextual knowledge, mobilised for instance through reliance on national experts, is not a guarantee of success. UHC policy processes are complex with many unknowns. For instance, we still do not know much on how to sequence steps towards UHC [61]. It may happen that, because of haste or oversimplification, experts do not see the limits of their toolbox, including analytical frameworks or generic political guidance developed by international agencies. This is particularly problematic if critical thinking is poorly developed among decision/policy-makers.

Scientists and experts can also be seen just as useful contributors to policy processes. This situation is encountered in some middle-income countries [62]; an issue then is that experts may be used instrumentally to justify certain choices. Scientists and experts may also be facing governments that know very well where they want to go but are in great need of advice on which path to take and are looking for experts willing to support them. In these situations, scientists and experts could be used as ‘facilitators’ since processes are country led. Examples include Rwanda or Ethiopia, which show strong leadership in their UHC policy choices and bring external partners to follow the path set by the government [63, 64]. The challenge for scientists and experts here is not to lose their independence vis-à-vis the government or the politicians and, sometimes, to push for more reflexivity. Lastly, one can imagine a Ministry or an actor that has some resources and capacities but appreciates external guidance on what to do and achieve. Scientists and experts could thus play the role of ‘producers of standards’, with the big challenge to produce high quality standards tailored to country needs — this is not obvious if the scientists or experts lack in-depth contextual knowledge. However, this could also include the use of normative frameworks produced by individual scientists or experts, or international agencies [53–55].

#### The case of reflexive learning

It seems to us that the international UHC community has so far paid little attention to this second mode of learning. In reality, it is taking place but it is not highlighted in the literature nor made explicit. In the context of UHC, a typical example of reflective learning could be the learning that emerges from what is coined “*démocratie sanitaire*” [health democracy] in the francophone system — that is, a process promoting citizen participation in health policies development and implementation through consultation, public debates and dialogue [65]. Actually, community actors are directly involved in policy processes and learning is collective, arising through the co-production of ideas and discussions. Another example of reflexive learning is deliberative processes bringing together various stakeholders to collectively reflect on complex issues to get better insights and suggest possible solutions [66, 67]. There are flat power relations between policy actors and no knowledge hierarchy — all types of knowledge are equally esteemed. Here, we can draw a parallel with the facilitation techniques using the rules of brainstorming — all the participants are equal, no idea is stupid and everyone participates. In the face of uncertainty, in a context of reforms, or in a situation where certain values and social norms must be questioned and new perspectives adopted, these open approaches based on dialogue, discussion, exchange of information and ideas are welcome rather than being an issue [65]. Indeed, they allow to gain mutual meaningful insights on issues at stake and there is room for serendipity as well as bold and innovative ideas.

Concretely, this learning mode seems little used in countries where decision-making is highly centralised. Indeed, reflexive learning in some way would constitute a kind of ‘endangerment’ as it involves losing some control over the policy process. The National Health Assemblies in Thailand, originating from the concept of the ‘Triangle that Moves the Mountain’ [68] (described below), which started in the early 2000s, and the Societal Dialogue for Health System Reform, launched in Tunisia in 2012 [69], are typical examples of situations where reflexive learning could occur. The societal dialogue in Tunisia aims to develop a health system more responsive to citizens’ expectations with a new mode of governance based on decentralisation and ‘health democracy’ [70]. However, as in any process where decisions have to be taken by many at the same time, this dialogue turned out to be quite complex [71].

#### The case of learning through bargaining

Learning through bargaining is probably the most overlooked mode of learning in the health policy literature. Yet, we think that it happens daily, as policy-makers constantly learn from their interaction with stakeholders. Such learning arises when there are exogenous or endogenous attempts to shift policy objectives or instruments. Learning will emerge from efforts done to reach an agreement. Examples include (1) the adoption (or not) of output-based financing mechanisms [72]; (2) the degree of autonomy to be granted to health facilities in terms of organisation, service delivery and use of resources [73]; (3) how health insurance funds should be collected [74], pooled, allocated and the benefit package designed [75]; (4) changes in the market structure of healthcare provision, including the promotion (or not) of the private sector — the so-called public–private partnerships [76]; and (5) more generally, the adoption of some reforms, laws and regulations in the health sector [77]. Learning through bargaining is also a distinctive feature of certain permanent mechanisms such as priority-setting and budgetary negotiations [78], discussions between donors and their countries counterpart to set up health policies [79] or, as part of the Global Fund, proposal developments for funding applications or Principal Recipients nominations by the Country Coordinating Mechanisms [80]. Another example of learning through bargaining is the ‘Triangle that Moves the Mountain’, a nice metaphor showing how sound interactions between key stakeholders in Thailand, namely researchers producing policy-relevant knowledge, civil society organisations and communities leading a social movement, and politicians providing required resources, have been able to promote social learning and yield major changes in a difficult context [81].

Learning through bargaining focuses on the preferences of stakeholders [47] and stems from a dialectical process. Government officials negotiating with each other (e.g. Ministry of Health with other ministries such as Social Welfare or Finance) and with external partners, civil society or unions to find out how to develop a coherent UHC policy are learning a lot. For instance, in Morocco, such learning occurred when several ministries with divergent views gathered around the same table to discuss RAMED (*Régime d’Assistance Médicale*; a health coverage scheme for the poor) options, each bringing their own knowledge and experience [82]. Bargaining is essential as UHC policies may not be consensual and require trade-offs or choices that are eminently political, often involving resources redistribution and disruption of power relations. There seems to be little guidance today on how to institutionalise this learning mode; knowledge mainly remains tacit, as ‘experience’ and can ‘evaporate’ quite quickly [49, 82].

#### The case of learning in the shadow of hierarchy

In the context of UHC, learning in the shadow of hierarchy emerges from the very exercise of public authority. It is therefore practiced by all health authorities, although in varying ways and quality. Hierarchical learning is particularly cherished by disease programmes and international agencies with an operational mandate such as UNICEF. Indeed, like epistemic learning, hierarchical learning is directed and prescriptive. It corresponds to the situation where an actor uses his/her/its ‘legitimate’, ‘coercive’ or ‘reward power’ [48] to purposively orient policy actions in a desired direction in order to enforce a policy or achieve specific goals or results. Let us mention that some people in a high hierarchical position can also be scientists or experts (e.g. scientists or experts being decision-makers or politicians) — we propose to consider these situations as learning in hierarchy instead of learning in epistemic contexts.

In any case, the quality of learning will depend on the compliance of the governed, the clarity on the roles of stakeholders and the effectiveness of instructions. Such learning, for example, is supported by field supervision visits, good monitoring and evaluation systems with adequate metrics to analyse policy actors’ performance, or annual policy reviews. It thus values deliverables and measurable results. Generally, knowledge is acquired both by the person holding authority who provides supervision or performs the monitoring and by the ‘street-level bureaucrats’ who make the effort to understand the instructions. Learning can be enhanced if the supervisor is able to take advantage of the lay knowledge of the grassroots actors and not necessarily believe that his/her own hierarchical position or seniority means superior knowledge.

In fact, hierarchical learning can be supported through the proper use of internal routine data. This is probably one of the great learning opportunities that remains minimally exploited for UHC [83]. Furthermore, like learning through bargaining, knowledge gained during the monitoring remains very tacit (embodied knowledge) and can get lost if, for example, a group of people is not stabilised at the head of UHC policy processes or if health workers are regularly deployed to other positions.

### The way forward

Several important points emerge from our research. It has shown that there are many ways to learn, materialised by the learning modes proposed by Dunlop and Radaelli. In-depth analyses of what mode(s) of learning is occurring, with whom, when, where, why, how, at what level and with what results, in relation to specific UHC processes in LMICs, deserve being empirically investigated and, we trust, will be the subject of future research. Further research exploring their triggers, constraints and pathologies [84] would also be relevant. For instance, as hindrances, tacit knowledge gained during learning can get lost if people are not stabilised at their positions or if the turnover is too high. Consequently, health authorities would lose learnings accumulated.

Learning for UHC naturally occurs. However, learning can also be organised or directed. Among ‘UHC promoters’, there seems to have been so far an operational bias towards epistemic learning, often under a teacher–learner model. It is undeniable that this learning mode demonstrates some effectiveness to tackle technical knowledge and capacity gaps in many LMICs, but this bias possibly stems also from power structures or unchallenged assumptions (e.g. donors knowing more than governments; academia knowing more than practitioners). In any case, there are probably missed opportunities — countries do not leverage the large array of learning situations that have great potential to spur their progress toward UHC. Today, too few ministries or technical departments are purposely investing in their own systemic learning capacities [10–12].

Beyond the illustrations we provided in this paper, applied work is needed to know more about what learning modes occur, when, where, how, at what level and under what circumstances. For instance, Akhnif et al. [82] already observed, in their case study on RAMED, that *"learning changes in nature across the different stages of the policy process"* — our study allows to deepen this subject matter by highlighting a grid to better categorise and analyse these different moments of learning. It would also be compelling to investigate what learning modes are mobilised or emerge at each stage of the strategic planning process, as proposed by WHO [85]. Furthermore, digitisation has created great potential for learning, both at the decision-making level and at the operational level, but this remains largely untapped [86] and further research is needed to unravel ways to better exploit this potential.

Other studies could also explore the contribution of different hybrid models, that is, varying degrees of mixtures of different learning modes. Indeed, learning modes are not mutually exclusive; they co-exist, occurring sometimes at the same time. A good example of a hybrid model is the ‘coaching and mentoring’ support provided by the Strategic Purchasing Africa Resource Centre (SPARC) to an Expert Panel of Kenya’s National Hospital Insurance Fund to comfort it as a strategic purchaser of health services [87]. This experience was rich in reflexive (through the engagement of various stakeholders), bargaining (to accommodate divergent opinions and reach consensus through formal and informal discussion channels) and epistemic (the mentor giving expert advice only when requested) learnings. Learning generated through pilot schemes, as was the case for performance-based financing with Rwanda [88], Health Equity Funds in Cambodia [89], the RAMED in Morocco [82], or user fees removal policies in Burkina Faso [90], is also an interesting case. Policy actors and experts knew what they were looking for but they also acknowledged that there were many unknowns. By combining their assets (public authority, ideas and experimental methods), they together constituted an original body of knowledge that could be used to inform decision-making and scale-up processes [91]. In the cases reported above, policy actors’ learning seemed to have combined at least the epistemic and reflexive modes, both enhanced by experimental action, as promoted by Garvin [92]. Similar hybrid learning is probably in application with the practice of study tours: there is an epistemic component (learning from another country with a more ‘advanced’ experience) and a reflexive component (since visitors, and possibly guests, informed by their own observations collectively reflect), before any experimentation or application back home.

## Conclusions

This paper aimed at illustrating the possibility and relevance of using the concept of ‘policy learning’ to analyse learning in UHC processes in LMICs. Dunlop and Radaelli’s framework allowed us to throw a new light on existing processes but also to widen the learning prospects that countries could tap into to advance their UHC agenda. The new perspectives highlighted in this article also echo implementation activities or research carried out by multiple actors involved in the field of learning for UHC — they could validate certain hypotheses, clarify grey areas and, above all, spark new reflections and ideas.

All in all, there is room for action and building countries’ systemic learning capacity for UHC. However, establishing an ambitious research and learning programme is crucial. Our contribution fits in this voluntarist perspective.

## Data Availability

Not applicable.

## Abbreviations

LMICs: Low- and middle-income countries
UHC: Universal health coverage
RAMED: Régime d’assistance médicale
